# Intraductal papilloma of ectopic breast tissue in axillary lymph node of a patient with a previous intraductal papilloma of ipsilateral breast: a case report and review of the literature

**DOI:** 10.1186/1746-1596-5-17

**Published:** 2010-03-12

**Authors:** Radan Dzodic, Boban Stanojevic, Vladimir Saenko, Masahiro Nakashima, Ivan Markovic, Gordana Pupic, Marko Buta, Momcilo Inic, Tatiana Rogounovitch, Shunichi Yamashita

**Affiliations:** 1 Institute for Oncology and Radiology of Serbia, 14 Pasterova, Belgrade 11000, Serbia; 2 Department of Molecular Medicine, Nagasaki University Graduate School of Biomedical Sciences, 1-12-4 Sakamoto, Nagasaki 852-8523, Japan; 3 Department of International Health and Radiation Research, Nagasaki University Graduate School of Biomedical Sciences, 1-12-4 Sakamoto, Nagasaki 852-8523, Japan; 4 Tissue and Histopathology Section, Division of Scientific Data Registry, Nagasaki University Graduate School of Biomedical Sciences, 1-12-4 Sakamoto, Nagasaki 852-8523, Japan

## Abstract

The presence of ectopic breast tissue in axillary lymph nodes (ALN) is a benign condition that must be differentiated from primary or metastatic carcinoma. Here we report a patient who underwent excision of enlarged ALN 10 years after she had received surgical treatment of ipsilateral breast for an intracystic intraductal papilloma (IDP). Histological examination of the removed ALN revealed that the proliferative lesion consisted of papillary and tubular structures lined by luminal cuboidal cells and a distinct outer layer of myoepithelial cells resembling IDP of the breast. Immunostaining with a set of immunohistochemical markers including AE/AE3, alpha-smooth muscle actin and p63 in combination with estrogen and progesterone receptors confirmed the diagnosis of ectopic IDP.

This case shows that even though benign proliferative change in ectopic breast tissue is an extremely rare phenomenon, this possibility should be taken into account for correct diagnosis.

## Background

Metastasis of breast cancer is most frequently found in axillary lymph nodes (ALN) [1]. In order to avoid overtreatment of patients, various benign lesions must be clearly differentiated from malignancy. Among the benign conditions, the presence of ectopic breast tissue (EBT) due to embryological displacement within ALN is an uncommon but well-recognized phenomenon [2]. Usually, the nodal EBT inclusion presents with a single or arranged in small clusters normal glandular structures sometimes associated with cystic changes. The presence of a benign proliferative lesion arising in ectopic duct epithelium is exceptionally rare [3]. In this report we present a case intraductal papilloma (IDP) in ALN of a patient who had been treated for IDP of the breast 10 years before.

## Case Presentation

**Figure 1:**
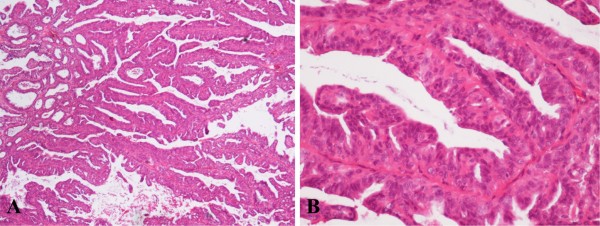
**Histological appearance of IDP of the left breast in low power field, ×40 (A)**. Two-cell pattern lined by luminal cuboidal cells and a distinct outer layer of myoepithelial cells under higher magnification, ×200 (B).

A 34 year-old woman previously operated for a solitary encapsulated papillary thyroid microcarcinoma in the right lobe of the gland, without familial history of breast or thyroid malignancy, was admitted to the Institute of Oncology and Radiology of Serbia, Belgrade, after feeling soreness at breast self-examination followed by a suspicious mammographic finding in the left breast. A lump measuring 30 × 30 × 20 mm in size was surgically removed. Grossly, the resected specimen featured a cyst (15 mm in diameter) with an intracystic proliferative lesion 10 × 10 × 8 mm in size. Histopatholological examination revealed an intracystic IDP without evidence of malignancy (Figure 1). No axillary lymphadenopathy was found at the time of surgery. Whereas no changes were seen in the patient's breast on ultrasound or mammography during follow-up, an enlarged lymph node became palpable in the left axilla 10 years later. A metastatic carcinoma in ALN was suspected and the patient was subjected to excisional biopsy.

**Figure 2:**
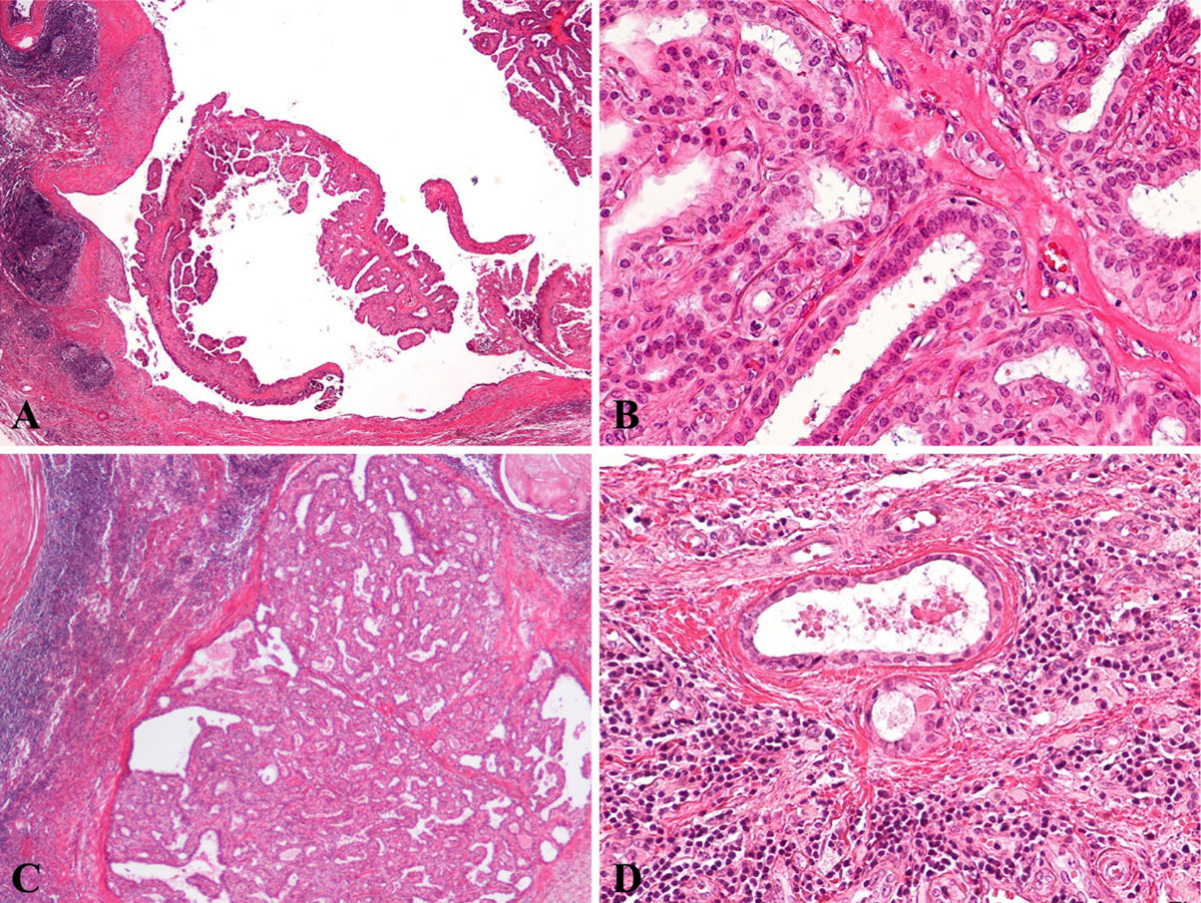
**Histological features of IDP in the left axillary lymph node**. Intracystic papillary proliferative lesion at low power, ×20 (A). Two-cell pattern lined by luminal cuboidal cells and a distinct outer layer of myoepithelial cells under higher magnification, ×200 (B). Intraductal papillary proliferative lesion, ×40 (C) and a small cluster of duct-like structures in the lymphoid tissue surrounding intracystic IDP, ×200 (D).

A lymph node sized 15 mm in diameter was removed. Histology revealed a proliferative epithelial lesion measuring 11 mm in the largest dimension in the cystic space of the node. The proliferative lesion consisted of papillary and tubular structures lined by luminal cuboidal cells and a distinct outer layer of myoepithelial cells which were very similar to IDP of the breast (Figure 2A and 2B). Apocrine metaplasia was found. No mitoses, necrosis or cells with atypical features were detected, suggesting the benign nature of the neoplasm. In addition, both the proliferative papillary lesion and small clusters of duct-like structures were also observed in the lymphoid tissue surrounding the cystic area (Figure 2C and 2D). Immunostaining with a series of antibodies (all from Dako, Carpinteria, CA, USA) was used to confirm histogenetic origin of tumor cells in ALN. Staining for AE/AE3 was diffusely positive in tumor epithelium and staining for alpha-smooth muscle actin and p63 was focally positive in myoepithelial cells. Neoplastic and ectopic duct-like epithelial cells were estrogen (ER) and progesterone receptors (PR)-positive cumulatively confirming their origin from the breast. As a result, the presented case was diagnosed as IDP of ectopic breast tissue in ALN on the basis of histopathological and immunohistochemical findings. Eight years after ALN excision, the patient was in excellent condition without any signs of recurrence.

## Discussion

The phenomenon of intranodal glandular inclusions in ALN attracts the attention of breast surgeons and pathologists since their presence may be mistaken for primary or metastatic carcinoma. At least several reasons for ALN enlargement through the accumulation of non-lymphoid tissue are known. These are the presence of nonneoplastic EBT, primary malignant or (rarely) benign EBT tumor, metastatic disease from distant primary sites of which the breast is the most probable, and previous surgical manipulations, principally on the breast, which may lead to the lymphatic dissemination of normal and/or tumor tissue. Of note, iatrogenic breast tissue inclusions in ALN, especially benign, usually remain microscopic [4-8]. Here we described a case of macroscopic, palpable IDP in ALN of a patient previously treated for IDP of ipsilateral breast. The neoplastic tissues found in ALN were ER- and PR-positive and featured a double layer of epithelial and myoepithelial cells indicating their mammary origin and the benign nature [9].

To the best of our knowledge, to date there are only two reports of IDP in ALN. The first described, without essential histological details, a case resembling papilloma of the breast in ALN [10]. The second provided a thorough analysis of a benign heterotopic papilloma in the sentinel node [11]. Based on extensive histological, immunohistochemical and molecular findings, the authors proposed that nodal neoplasm could arose from a benign implant of breast papilloma which was removed at mastectomy after several diagnostic fine-needle aspiration biopsies and surgical manipulations, or it might have developed *de novo *from the ectopic breast inclusion of the lymph node.

In the case that we described, we also could not unambiguously distinguish whether nodal IDP developed as a result of tissue dissemination due to the previous breast surgery or from EBT. Nevertheless, we are rather inclined towards the latter since both IDP and normal duct-like structures in lymphoid tissue outside the major lesion were observed (Figure 2). Their presence may be suggestive of preexisting EBT, some parts of which underwent benign neoplastic change. Whatever the cause, history of previous breast surgery may provide important information for enlarged ALN diagnosis.

The case presented here provides additional evidence that EBT can undergo proliferative changes in ALN which may lead to clinical suspicion for cancer. These changes, however, may not necessarily be malignant as this case shows, and surgeons and pathologists need to be aware of this rare but possible scenario. Distinctive morphological features such as two-cell pattern and a set of highly informative immunohistochemical markers including AE/AE3, alpha-smooth muscle actin and p63 in combination with ER and PR enable reliable diagnosis of ectopic IDP allowing to avoid overtreatment and improving patient's quality of life.

## Conclusion

The presence of ectopic breast tissue in axillary lymph nodes (ALN) is a benign condition that must be differentiated from primary or metastatic carcinoma. Here we report a patient who underwent excision of enlarged ALN 10 years after she had received surgical treatment of ipsilateral breast for an intracystic intraductal papilloma (IDP). This case and the review of similar cases presented in the literature show that even though benign proliferative change in ectopic breast tissue is an extremely rare phenomenon, the possibility of such disease should be taken into account for correct diagnosis.

## Competing interests

The authors declare that they have no competing interests.

## Authors' contributions

The patient was examined and operated by RD, IM, MI and MB; these authors are responsible for the post-operative care, follow-up and clinical information. MN and GP performed histopathological examination. BS and VS were responsible for the main conception and review of the literature. This manuscript was drafted by BS and VS, and then critically reviewed by TR and SY. All authors have read and approved the final manuscript.
